# Cerebral Dopamine Neurotrophic Factor (CDNF) Has Neuroprotective Effects against Cerebral Ischemia That May Occur through the Endoplasmic Reticulum Stress Pathway

**DOI:** 10.3390/ijms19071905

**Published:** 2018-06-29

**Authors:** Geng-Lin Zhang, Li-Hong Wang, Xing-Yu Liu, Ya-Xuan Zhang, Meng-Yang Hu, Lin Liu, Yuan-Yuan Fang, Yu Mu, Yan Zhao, Shu-Hong Huang, Ting Liu, Xiao-Jing Wang

**Affiliations:** 1 Department of Cell Biology and Neurobiology, School of Basic Medicine, Shandong University, No. 44 Wenhua Xi Road, Jinan 250012, China; zgelin@163.com (G.-L.Z.); wanglihong1202@163.com (L.-H.W.); 18609831660@163.com (X.-Y.L.); 17860624912@163.com (Y.-X.Z.); m4water@163.com (M.-Y.H.); b1770093-liulin@sjtu.edu.cn (L.L.); doctorfyy98@163.com (Y.-Y.F.); mumumumu9@126.com (Y.M.); zhaoyan623@yeah.net (Y.Z.); shuhonghuang@126.com (S.-H.H.); liuting@sdu.edu.cn (T.L.); 2 Key Laboratory for Biotech-Drugs Ministry of Health and Key Laboratory for Rare & Uncommon Diseases of Shandong Province, Shandong Medicinal Biotechnology Center, Shandong Academy of Medical Sciences, Jinan 250062, China

**Keywords:** CDNF, stroke, oxygen–glucose depletion, neuroprotection

## Abstract

Cerebral dopamine neurotrophic factor (CDNF), previously known as the conserved dopamine neurotrophic factor, belongs to the evolutionarily conserved CDNF/mesencephalic astrocyte-derived neurotrophic factor MANF family of neurotrophic factors that demonstrate neurotrophic activities in dopaminergic neurons. The function of CDNF during brain ischemia is still not known. MANF is identified as an endoplasmic reticulum (ER) stress protein; however, the role of CDNF in ER stress remains to be fully elucidated. Here, we test the neuroprotective effect of CDNF on middle cerebral artery occlusion (MCAO) rats and neurons and astrocytes treated with oxygen–glucose depletion (OGD). We also investigate the expression of CDNF in cerebral ischemia and in primary neurons treated with ER stress-inducing agents. Our results show that CDNF can significantly reduce infarct volume, reduce apoptotic cells and improve motor function in MCAO rats, while CDNF can increase the cell viability of neurons and astrocytes treated by OGD. The expression of CDNF was upregulated in the peri-infarct tissue at 2 h of ischemia/24 h reperfusion. ER stress inducer can induce CDNF expression in primary cultured neurons. Our data indicate that CDNF has neuroprotective effects on cerebral ischemia and the OGD cell model and the protective mechanism of CDNF may occur through ER stress pathways.

## 1. Introduction

Neurotrophic factors (NTFs) are an important group of secreted proteins that promote the survival, growth, and differentiation of neural cells. NTFs not only regulate the survival of neurons during development but also have neuroprotective roles, such as inhibiting the death of mature neurons after injury, promoting the repair of neurons and axon regeneration, and regulating the plasticity of synapses and the transmission of neurotransmitters. NTFs include the following families: neurotrophins, which include nerve growth factor (NGF), brain-derived neurotrophic factor (BDNF), neurotrophin-3 (NT-3) and NT-4; glial cell line-derived neurotrophic factor (GDNF) family ligands (GFLs) GDNF, neurturin (NRTN), artemin (ARTN), and persephin (PSPN); and neuropoietic cytokines (also referred to as the interleukin-6 (IL-6) family) [1]. Because NTFs have many beneficial effects on neurons, they are potential factors for the treatment of neurodegenerative diseases and neuronal trauma. Although GDNF and NRTN are two highly potent NTFs in the treatment of Parkinson’s disease (PD), the outcomes of clinical trials are not very successful [1,2,3]. To search for and study the novel NTFs that could offer new therapeutic approaches for the treatment of neurodegenerative diseases is urgently needed. 

CDNF belongs to a novel, evolutionarily conserved CDNF/MANF family that first showed neurotrophic activities in dopaminergic neurons [4,5,6]. CDNF can significantly reduce amphetamine-induced ipsilateral turning behavior in rats and can almost completely rescue dopaminergic tyrosine-hydroxylase-positive cells in the substantia nigra [5]. A characteristic feature of CDNF proteins is the eight cysteine residues, the spacing of which is conserved from vertebrates to invertebrates [4,5,7]. Human CDNF exhibits a 59% amino acid identity with human MANF. The structure of CDNF includes two domains: a saposin-like N-terminal domain and a presumably unstructured C-terminal domain with an intradomain cysteine bridge in a ^32^CRAC^135^ motif. At the very end of the C-terminus, the CDNF has lysine-threonine-glutamicacid-leucine (KTEL) sequences, which closely resemble the classical ER retention signal (KDEL) [8,9]. This suggests that CDNF may have important functions in the endoplasmic reticulum (ER). 

ER stress is caused by the accumulation of unfolded proteins in the ER that induce the unfolded protein response (UPR). The UPR pathway counteracts ER stress by increasing protein folding capacity and the degradation of misfolded proteins in the cell. If ER stress persists or is activated excessively, cell apoptosis will eventually be initiated [10,11]. Many studies have shown that family member MANF is an endoplasmic reticulum (ER) stress protein [9,12,13,14,15,16,17,18]. Unlike MANF, expression of CDNF was not upregulated by tunicamycin-induced ER stress in U2OS cells, suggesting that CDNF may be expressed constitutively [9]. However, little is known about the role of CDNF in ER stress currently. An ER stress response element in the CDNF promoter region has not been reported.

ER stress has been shown to be involved in brain ischemia [19,20,21,22,23,24]. In this study, we investigated both the expression profile of CDNF via cerebral ischemia and the protective effect of CDNF on ischemic brain injury. The expression of CDNF in cultured primary neurons treated with ER stress-inducing agents and the protective effects of recombinant human CDNF on an oxygen–glucose depletion (OGD) cell model was also investigated. 

## 2. Results

### 2.1. Pretreatment with CDNF Significantly Reduced the Cerebral Infarction Volume

**Figure 1:**
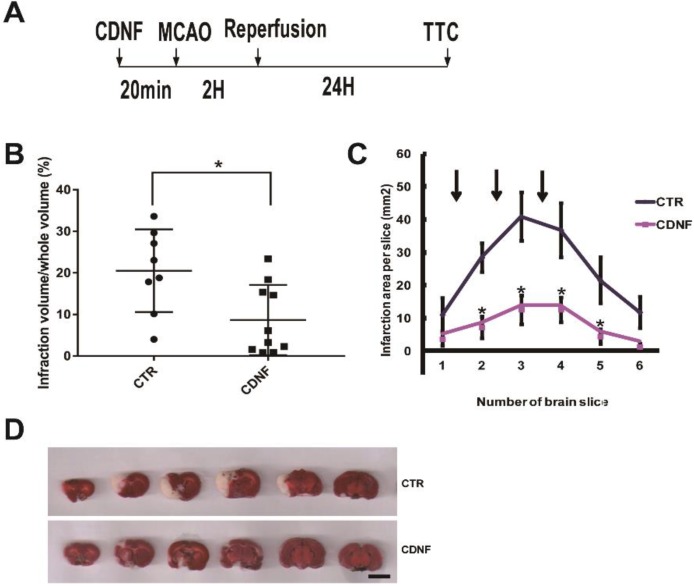
The local cortical CDNF pretreatment significantly reduced the cerebral infarction volume induced by MCAO. (**A**) Experimental scheme for the MCAO rats. CDNF protein or vehicle was administered intracerebrally into three cortical sites approximately 20 min before MCAO. The left middle cerebral artery (MCA) was occluded with a nylon filament for 120 min, and the filament was withdrawn to allow for 24 h of reperfusion. Then, the brains were removed and sliced into 2.0-mm thick sections for triphenyltetrazolium chloride (TCC) staining. (**B**) Pre-treatment with CDNF (6 μg) significantly decreased the infarct volume in the MCAO rats (control group, *n* = 8; CDNF group, *n* = 10). * *p* < 0.05 versus CTR. (**C**) The comparison of the infarction area per slice in each of the groups treated with CDNF (*n* = 10) and PBS (*n* = 8). The CDNF group significantly reduced the infarction area. Arrows represent the sites of injection. * *p* < 0.05 versus CTR. (**D**) TTC staining of brain sections. From left to right, the representative images were placed in order from the anterior to the posterior portion of the brain. Scale bar = 10 mm.

Rats were pretreated with phosphate buffer saline (PBS) (*n* = 8) or with 6 μg of CDNF (*n* = 10) (Figure 1A). The effects of CDNF on brain infarct volume are shown in Figure 1B,D. A significantly smaller infarct volume was observed in the CDNF pretreatment group than in the PBS treatment group after a 24-h reperfusion (8.67 ± 8.45% versus 20.53 ± 9.96%). To further examine the topographic relationship of protection, the area of infarction in each slice was compared in animals receiving with 6 μg of CDNF or PBS. A significantly smaller infarction was found in animals treated with 6 μg CDNF than in those treated with PBS, especially in the second slice (7.24 ± 3.32% versus 28.44 ± 4.33%), third slice (12.62 ± 4.39% versus 40.92 ± 7.40%), fourth slice (12.63 ± 3.83% versus 36.81 ± 8.15%), and fifth slice (4.47 ± 2.18% versus 21.52 ± 6.98%) shown in Figure 1C.

### 2.2. Pretreatment with CDNF Promoted Behavioral Recovery after MCAO

**Figure 2:**
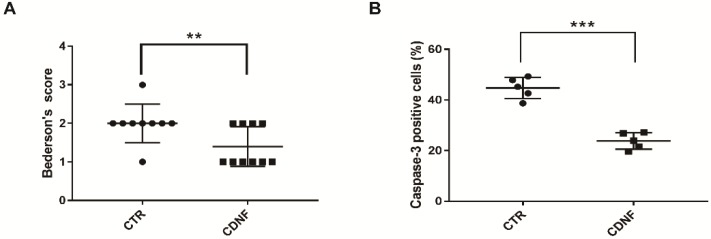
The local cortical CDNF pretreatment significantly reduced neurological scores and cell apoptosis in MCAO rats. (**A**) CDNF pretreatment significantly reduced the Bederson score (control group, *n* = 8; CDNF group, *n* = 10). ** *p* < 0.01 versus CTR by Mann-Whitney u test. (**B**) Quantitative analysis of the number of caspase-3 positive cells in the cortexes (control group, *n* = 5; CDNF group, *n* = 5). *** *p* < 0.001 versus CTR. Student’s *t*-test. Data are expressed as the mean ± SD.

Neurological deficits were assessed after 2 h of ischemia/24 h of reperfusion. The Bederson score was 2.0 ± 0.17 in the PBS-treated animal groups. The other group pretreated with 6 μg of CDNF showed a significant reduction in the Bederson score compared to the MCAO group (1.4 ± 0.16, Figure 2A). This result suggested that pretreatment with 6 μg of CDNF could reduce the animals’ neurological deficits and promote behavioral recovery.

### 2.3. Pretreatment with CDNF Reduced the Number of Caspase-3 Positive Cells in the Cerebral Cortex after MCAO

Cleaved caspase-3 is the main marker for apoptotic cells. MCAO caused cell apoptosis or necrosis in the ischemic cerebral cortex. Caspase-3 positive cells (44.76 ± 1.88%) were found in the cerebral cortex of the MCAO rats. There were significantly fewer caspase-3 positive cells in the other group pretreated with 6 μg of CDNF (23.84 ± 1.46%, Figure 2B). These data implied that CDNF had neuroprotective effects on the cell apoptosis caused by ischemia in the MCAO rats. In order to identify the cytotypes of caspase-3 positive cells in the cortex, we performed the double immunofluorescent staining with antibodies against caspase-3, NeuN (a marker for neurons), or GFAP (a marker for astrocytes). The data showed the caspase-3 positive cells were NeuN-positive cells but not GFAP-positive cells). This suggested the apoptotic cells in the ischemic cortex are neurons.

### 2.4. Pretreatment with CDNF Increased the Viability of Neurons and Astrocytes Treated with OGD

**Figure 3:**
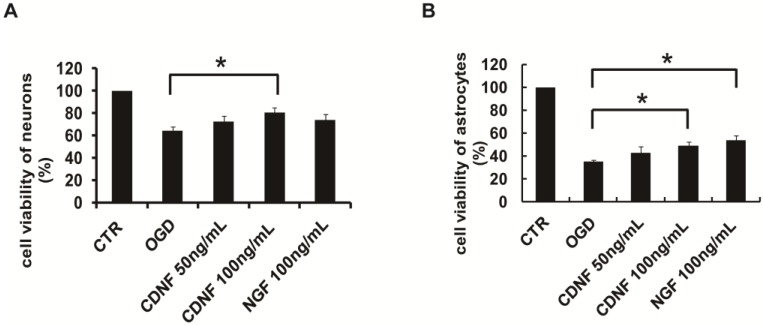
The effects of CDNF on cultured primary neurons and astrocytes during OGD treatment. (**A**) Preincubation of CDNF (100 ng/mL) significantly increased the cell viability of primary neurons that was caused by OGD. (**B**) Preincubation of CDNF (100 ng/mL) significantly increased the cell viability of primary astrocytes that was caused by OGD. NGF (100 ng/mL) was used as a positive control. * *p* < 0.05 CDNF (100 ng/mL) or NGF (100 ng/mL) versus OGD group tested by one-way Analysis of Variance (ANOVA). Three independent experiments were performed and the reported data represent the mean of the three experiments.

To mimic ischemic conditions in vitro, we set up an OGD model of neurons and astrocytes. To further test the protective effect of CDNF, the cells were pretreated with recombinant human CDNF at different concentrations for 1 h before the OGD. After 2 h of OGD and 24 h reoxygenation, the viability of the neurons decreased about 64.14 ± 4.09%, as assessed by the impairment in mitochondrial function (3-(4,5-Dimethylthiazol-2-yl)-2,5-diphenyltetrazolium bromide, MTT test) (Figure 3A). When exposure to 50 ng/mL of CDNF, the viability of the neurons increased to approximately 72.81 ± 4.97%, and the viability approximately 80.69 ± 4.21% at the concentration of 100 ng/mL. As a positive control, the viability increased to 73.59 ± 5.49 treated by 100 ng/mL of NGF (Figure 3A). CDNF (100 ng/mL) significantly increased the viability of neurons compared to those in the OGD group. After 24 h of OGD and 24 h reoxygenation, the viability of astrocytes decreased by approximately 35.08 ± 1.88% (Figure 3B). The viability increased by approximately 42.40 ± 5.91% when exposing to 50 ng/mL of CDNF, and the viability increased by approximately 48.88 ± 3.78% at the concentration of 100 ng/mL. The viability of the astrocytes increased to 53.60 ± 4.41 (Figure 3B) when treated by 100 ng/mL of NGF. Recombinant human CDNF protected astrocytes against OGD-induced injury in a dose-dependent manner. The viability of astrocytes treated by 100 ng/mL of CDNF and NGF was significantly higher than the OGD group. This suggested that CDNF can protect neurons and astrocytes against OGD-induced injuries in a manner dependent on the amount of CDNF. 

### 2.5. The Expression of CDNF in the Ischemic Cerebral Cortex

**Figure 4:**
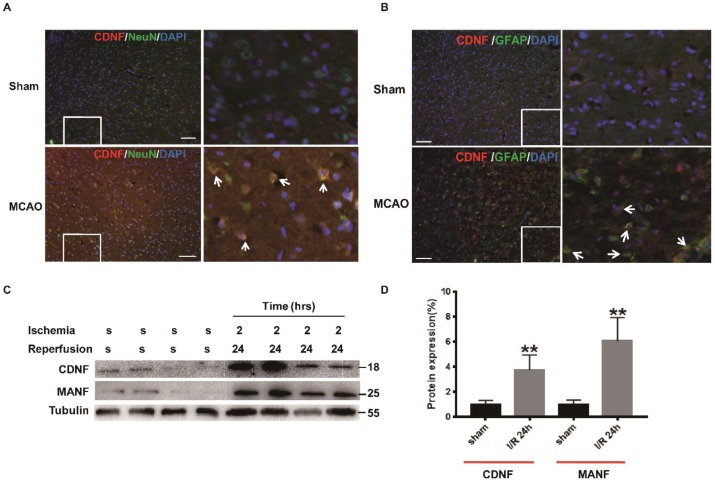
The expression of CDNF in the ischemic cerebral cortex. (**A**) CDNF (red)/NeuN (green) double labeling in the ipsilateral cortexes. DAPI (blue) labeled all the nuclei. Scale bar = 45 μm. The insets show photomicrographs of the cortex at higher magnification. Arrows point to the CDNF^+^/NeuN^+^ cells. (**B**) CDNF (red)/GFAP (green) double labeling in the ipsilateral cortexes. DAPI (blue) labeled all the nuclei. Scale bar = 45 μm. The insets show photomicrographs of the cortex at higher magnification. Arrows point to the CDNF^+^/GFAP^+^ cells. (**C**) The representative Western blotting to show the expression of CDNF. The samples of the ischemic penumbra were collected at I/R 24 h. Tubulin was used as a loading control. S, sham; MANF as the positive control. (**D**) Protein levels were quantified as relative levels compared to Tubulin, three independent experiments were performed and the reported data represent the mean of the three experiments, Student’s *t*-test, ** *p* < 0.01.

Next, we explored the mechanism of neuroprotection via CDNF. Focal cerebral ischemia has been shown to be an in vivo ER stress model, such that ischemia induces accumulation of immature proteins in the ER. First, the characteristics of ischemia-induced CDNF expression in the cerebral cortex were investigated using immunohistochemical assays and Western blotting in MCAO rats. It was found that more CDNF-positive cells appeared in the peri-infarct tissue following 2 h of ischemia and 24 h of reperfusion compared with the sham group (Figure 4A,B). To investigate the subpopulations of CDNF-positive cells, we performed the double immunofluorescent staining with antibodies against CDNF, NeuN (a marker for neurons) or GFAP (a marker for astrocytes). Some CDNF-positive cells were colocalized with NeuN-positive cells or GFAP-positive cells (Figure 4A,B). We have quantified the co-localization of CDNF with NeuN or GFAP. The CDNF^+^/NeuN^+^ cells in I/R 24 h ischemia group increased significantly than in the sham group (10.41 ± 8.35% versus 0.65 ± 0.58%). The CDNF^+^/GFAP^+^ Cells also increased significantly than in the control group (27.65 ± 8.95% versus 0.24 ± 0.09%). Western blotting was used to assess the expression of CDNF following 2 h of ischemia and 24 h of reperfusion. The level of CDNF and MANF in the cerebral cortexes of ischemic rats significantly higher compared to sham rats at 2/24 h (I/R) (Figure 4C,D and Figure S1). These results indicated that CDNF expression was induced by focal ischemia as MANF. 

### 2.6. The Expression of CDNF in Cultured Neurons Treated with an ER Inducer

**Figure 5:**
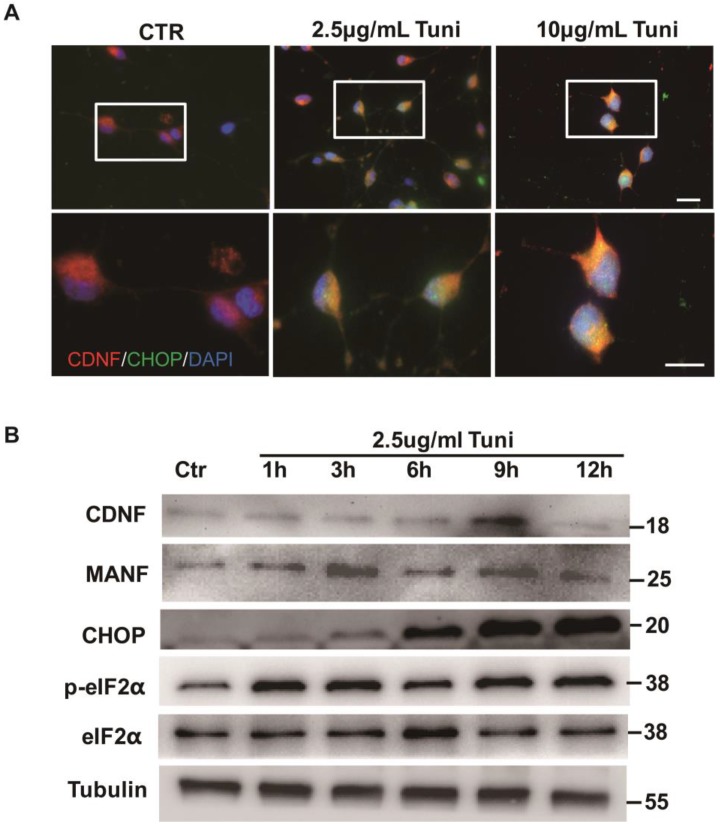

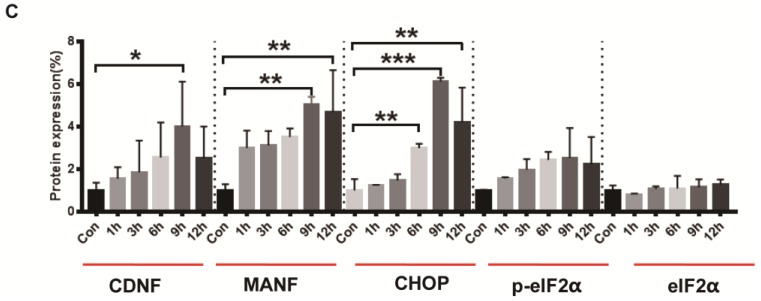
The expression of CDNF in cultured primary neurons treated with an ER inducer. (**A**) CDNF (red)/CHOP (green) double labeling in the primary neurons. The cells were treated with vehicle or tunicamycin (2.5 or 10 μg/mL) for 4 h before staining. DAPI (blue) labeled all the nuclei. Scale bar = 45 μm. The insets show photomicrographs of the primary neurons at higher magnification. Scale bar=25μm. (**B**) The representative Western blotting to show the expression of CDNF and ER markers. The primary neurons were treated with 2.5 μg/mL of tunicamycin for 1, 3, 6, 9 and 12 h before being collected. Tubulin was used as a loading control; MANF as the positive control. (**C**) Protein levels were quantified as relative levels compared to Tubulin, three independent experiments were performed and the reported data represent the mean of the three experiments, one-way ANOVA, * *p* < 0.05, ** *p* < 0.01 and *** *p* < 0.001.

To further confirm whether CDNF is an ER stress protein, we used primary neurons from E18 rats. The neurons were treated with the ER stress inducer tunicamycin (2.5 mg/mL or 10 mg/mL) for 4 h. C/EBP homologous protein (CHOP) is a stress-inducible nuclear protein that is usually used as a marker of ER stress [25,26]. To investigate whether CDNF is sensitive to ER stress, the expression levels of CDNF and CHOP were determined by using immunofluorescent staining. It was found that the number of CHOP-positive cells increased compared with those of the control group. The CHOP-positive cells became brighter as the dose of tunicamycin increased. However, the CDNF-positive cells exhibited no significant change in response to treatment with tunicamycin, even at high doses (Figure 5A). The neurons were treated with 2.5 mg/mL tunicamycin for 1, 3, 6, 9, and 12 h. Then immunoblotting with individually antibodies at the designated time points were performed. Western blotting showed that the expression of CHOP significantly increased by treatment with 2.5 mg/mL of tunicamycin for 6, 9, and 12 h compared with the control. The expression of eukaryotic initiation factor 2 alpha (eIF2α) and the phosphorylation of the eIF2α were kept unchanged from treatment for 1 to 12 h. The expression of MANF significantly increased after treatment for 9 and 12 h as the positive control. The expression of CDNF significantly increased after treatment for 9 h (Figure 5B,C and Figure S2). These findings suggested that ER stress can induce CDNF expression in primary cultured neurons. It seems that, like MANF, CDNF might be an ER stress protein. 

## 3. Discussion

In the present study, we evaluated the protective effects of CDNF on both MCAO rats and OGD-treated neurons and astrocytes. The present data provided the first evidence that CDNF can significantly reduce infarct volume, reduce apoptotic cells and improve motor function in MCAO rats, as well as increase cell viability in response to treatment with OGD. Thus, the expression profile of CDNF in ischemic brain and the primary neurons treated with ER stress-inducing agents suggested that the protective mechanism of CDNF may occur via ER stress.

The neuroprotective effect of CDNF was first identified in a 6-OHDA rat model of Parkinson’s disease [5]. CDNF (3 μg) significantly reduced the rotational behavior of the rats and increased the number of TH-positive cells in the substantia nigra pars compacta [5]. However, CDNF (100 ng/mL) did not promote the survival of P1 mouse superior cervical ganglion (SCG) sympathetic neurons or E14 and E15 mouse dorsal root ganglion (DRG) sensory neurons in culture. CDNF (0.1–100 ng/mL) had no effect on the survival of E14 rat motoneurons in vitro [5]. Subsequently, neuroprotective and neurorestorative studies of CDNF were performed in a 6-OHDA rat model or a mouse MPTP model of Parkinson’s disease [27,28]. Our data provided the first evidence of the neuroprotective effects of CDNF against cerebral ischemia in MCAO rats. Local administration of recombinant human CDNF to the cerebral cortex before MCAO could significantly reduce infarct volume, reduce the number of caspase-3 positive cells and improve motor function. MANF, which also belongs to a novel evolutionarily conserved protein family, was reported to have the same neuroprotective effect after ischemic brain injury [17,26,29]. These findings imply that CDNF and MANF can be considered potential treatments for ischemic injury.

We then investigated the neuroprotective effect of CDNF on the OGD cell model. Our data showed that CDNF had a neuroprotective effect on neurons and astrocytes against OGD-induced injury that was related to the amount of CDNF. It was reported that MANF alleviated OGD-induced astrocytic damage and inflammatory cytokine secretion by suppressing ER stress in rat primary astrocytes [30]. The anti-inflammatory property of CDNF was reported in lipopolysaccharide-induced microglia [31]. Inflammation is an important contributing factor to central nervous system (CNS) injury. Astrocytes play an important role in the processes of inflammatory responses in the CNS by secreting proinflammatory and anti-inflammatory cytokines, chemokines, and trophic factors [32,33]. Ischemic injury in the brain stimulates damage to astrocytes, which, in turn, release those factors. Overexpression of CDNF in primary astrocytes has shown the potential to alleviate cell damage induced by tunicamycin and proinflammatory cytokine secretion [31]. Astrocyte-derived factors are important in both neuronal survival and repair [34]. Astrocytic damage and the inflammation caused by ischemic injury are crucial pathological processes in the CNS. The neuroprotective role of CDNF or MANF in ischemic brain injury may reduce the damage to astrocytes, thus, directly or indirectly aiding the survival of neurons. Therefore, CDNF may be a potential novel agent for neuroprotective and neuroinflammatory treatments for stroke.

Accumulating evidence has suggested that MANF is an ER stress response protein that is able to protect cells against ER stress-induced cell death in vitro [1,9,12,13,18]. In vivo, transient upregulation of MANF mRNA was found in the adult rat brain after status epilepticus and global forebrain ischemia [16]. MANF protein was upregulated in cortical neurons after cortical ischemia induced by MCAO in rats [9,26]. MANF expression was induced in MCAO rats at the early stage of ischemia, starting from 2 h of ischemia/2 h reperfusion [26]. Our data showed that the expression of CDNF was upregulated in the peri-infarct tissue at 2 h of ischemia/24 h reperfusion in the MCAO rats. In addition, the expression of CDNF was upregulated in the primary neurons treated with an ER inducer. Because the structure of human CDNF has 59% amino acid identity with human MANF, both have C-terminal domains with an intradomain cysteine bridge in a CXXC motif, suggesting that CDNF and MANF may be involved in protein folding in the ER. Like MANF, these findings suggested that CDNF may be an ER stress response protein too.

In conclusion, this study was the first to reveal that CDNF can reduce both infarct volume and the number of apoptotic cells and improve motor function in MCAO rats. CDNF also increased the viability of cells treated with OGD. Our results provided new evidence of the neuroprotective effects of CDNF in cerebral ischemia and cells, which may occur through ER stress. Further studies exploring the mechanisms underlying the effects of CDNF on stroke may lead to a better understanding of the potential for therapies.

## 4. Materials and Methods

### 4.1. Experimental Materials

In the experiments, adult male Sprague-Dawley (SD) rats weighing 280–350 g were used and obtained from Vital River Laboratories (Beijing, China). The rats were kept at 12 h light/dark cycles. The ethics committee of the School of Medicine of Shandong University approved this study. All procedures were approved by the Ethics Committee on Animal Experiments of Medical School of Shandong University (No. 201502070, 7 June 2015).

### 4.2. Intracerebral Injections and Middle Cerebral Artery Occlusion (MCAO)

First of all, animals were subjected to treatment with intracerebral administration of CDNF (*n* = 8) or vehicle (PBS, *n* = 10). After 20 minutes, MCAO were performed to all animals for 2 h following reperfusion 24 h. Recombinant human CDNF protein (R&D Systems Inc., Minneapolis, MN, USA) was dissolved in PBS. The 6 μg of CDNF protein or vehicle was administered intracerebrally into three cortical sites, as previously described [16,35]. Two microliters of CDNF solution (3 μg/μL) or PBS was injected at a rate of 1 μL/min at each site. The needle was retained in place for 5 min after injection. 

Animals were anesthetized with 10% chloral hydrate by intraperitoneal injection (3 mL/kg body weight). Firstly it need to identify and isolate the left common carotid arteries through a ventral midline cervical incision. A nylon filament (0.28 mm in diameter) with a rounded tip was used. It was introduced from the common carotid artery lumen into the internal carotid artery to block the origin of the left middle cerebral artery (MCA). The left MCA was occluded with the filament for 2 h, then the filament was withdrawn to allow for 24 h of reperfusion. A left neck incision was also made to expose the arteries in the sham group. However, the nylon thread was not inserted into the internal carotid artery. During the recovery from the anesthesia, the animals were returned to their home cages. 

### 4.3. Examination of Neurological Deficits

The behavioral assays were scored as previously described [36]. After the 24-h reperfusion, the rats were examined. The rats were suspended 20–30 cm above the testing table and were scored. 

### 4.4. Evaluation of Infarct Volume

After 24 h reperfusion, the infarction area was measured by TTC staining. Rats were decapitated and the brains were removed carefully and sliced into 2.0-mm thick coronal sections using an acrylic rat brain block. The fresh brain slices were incubated in 2% TTC solution (Sigma-Aldrich, St Louis, MO, USA) for 30 min at 37 °C and were then transferred to a 4% paraformaldehyde solution for fixation. After staining, digital photographs were taken. Areas of the infarction in each slice were measured in the NIH’s ImageJ software, version 1.46. The area of infarction in each section was the average of the sum of two sides. The volume of infarction for each animal was obtained from the product of the average slice thickness (2 mm) and summing the infarct areas in all brain slices. The results were represented as the percentage of the total volume.

### 4.5. Immunohistochemistry

Serial sagittal or coronal (40 μm) sections were cut with a cryostat and stored at −80 °C. For the immunofluorescent staining, the sections were incubated overnight with primary antibody at 4 °C. The primary antibodies used as followed: rabbit polyclonal anti-CDNF (1:500; Sigma-Aldrich, St Louis, MO, USA); mouse monoclonal anti-NeuN (1:500; Millipore, Billerica, MA, USA); mouse polyclonal anti-GFAP (1:1000; Santa Cruz Biotechnology, Santa Cruz, CA, USA); rabbit monoclonal anti-cleaved caspase-3 (1:500; Cell Signaling Technology, Danvers, MA, USA). The secondary antibodies that were used were Alexa Fluor 488-conjugated IgG (1:1000; Invitrogen, Carlsbad, CA, USA) and Alexa Fluor 594-conjugated IgG (1:1000; Invitrogen, Carlsbad, CA, USA). Vectashield (Vector, Burlingame, CA, USA) was used to mount the sections. The nuclei of cells were counterstained with 4,6-diamidino-2-phenylindole (DAPI). 

### 4.6. Microscopy Analysis

Serial 40-μm coronalsections were cut. Every sixth section from one brain was collected for staining. To quantify the caspase-3 staining, immunofluorescence was visualized and imaged using a Nikon 80i light microscope (Nikon Instech Co., Ltd., Tokyo, Japan) equipped with a CCD camera. NIH ImageJ was used for blinded cell counting of images. The number of caspase-3 positive cells was counted in the penumbra region of the cerebral cortex per rat in each group (*n* = 5). The same penumbra region of cerebral cortex in each rat was selected for counting.

### 4.7. Primary Cell Culture

Cultures of hippocampal neurons from embryonic day-18 (E18) rats were prepared as described previously [37]. In brief, embryos were removed from the pregnant Sprague Dawley rats, then the hippocampi were dissected and dissociated with 0.05% trypsin-EDTA for 10 min at 37 °C and gently agitated with a sterile, fire-polished glass Pasteur pipette in a 1:1 mixture of DMEM/F12 (Invitrogen) plus with 10% fetal bovine serum (Invitrogen). The neurons were cultured in 60-mm dishes (Corning, NY, USA) coated with 0.1 mg/mL poly-d-lysine (Sigma-Aldrich). The culture medium was Neurobasal medium (Invitrogen) supplemented with 2% B27 and 0.5 mM glutamine. An incubator with 5% CO_2_ and 37 °C was used for the culture.

Primary astrocytes were prepared from newborn rat pups. Briefly, neonatal rats were anesthetized and decapitated. The cerebral cortices were removed and cut into small cubes (<1 mm^3^). Trypsin (0.25%, Sigma-Aldrich) was used to digest the tissues for 30 min at 37 °C and trypsinization was terminated by the addition of Dulbecco’s-modified Eagle’s medium (Invitrogen) containing 10% fetal bovine serum (Invitrogen). Then mechanical trituration was followed with a flame-polished Pasteur glass pipette. The cultures were incubated at 37 °C in a humidified atmosphere of 5% CO_2_. The culture medium was exchanged on day 3 and subsequently every 3–4 days. Astrocytes were passaged after the cultures reached confluence.

### 4.8. OGD Model

In order to mimic ischemic conditions, OGD was used on the cells in vitro. The neurons cultured for 7 days and the astrocytes passaged 3 times were treated by OGD. Before OGD, the cells were pretreated with PBS (control) and recombinant human CDNF (R&D Systems) at the concentration of 50 or 100 ng/mL for 1 h. CDNF was dissolved in PBS (pH 7.4). The culture medium was replaced by deoxygenated glucose-free Earl’s balanced salt solution (EBSS). T EBSS solution included (in mM) 0 glucose, 21 NaHCO_3_, 120 NaCl, 5.36 KCl, 0.33 Na_2_HPO_4_, 0.44 KH_2_PO_4_, 1.27 CaCl_2_, and 0.81 MgSO_4_ [38]. Cultures were then treated for 2 or 24 h in a hypoxic environment of 5% CO_2_, 1% O_2_, and 94% N_2_ at 37 °C. Cells were washed by PBS two times to terminate OGD. Then the culture medium was replaced and returned the cultures to a normoxic incubator maintained at 37 °C and 5% CO_2_ for 24 h reoxygenation. 

### 4.9. MTT

Cell viability was detected by MTTassay. Neurons and astrocytes were plated into 96-well culture plates at a density of 0.5 × 10^5^ cells/mL. Per well in triplicate was added 200 μL of culture medium. When reaching 70–80% confluency, the cells were pretreated with CDNF for 1 h. OGD treatment was followed for an additional 2 or 24 h. After 24 h reoxygenation, 20 μL MTT solution (5 mg/mL, Sigma-Aldrich) was added to each well and incubated at 37 °C for 4 h. The culture medium was aspirated and 200 μL of dimethyl sulfoxide was added. The absorbance value was measured at 490 nm with a Model 680 Microplate Reader (Bio-Rad Laboratories, Hercules, CA, USA).

### 4.10. Immunocytochemistry

Neurons were cultured on coverslips in 24-well plates. Cells were treated with 2.5 or 10 μg/mL of tunicamycin (Sigma-Aldrich) for 4 h. Cells were washed in PBS three times. 4% formaldehyde was used to fix for 10 min. Permeabilization was used by 0.2% Triton X-100 for 10 min at room temperature. After PBS washing, cells were blocked in 1% BSA in PBS for 20 min at room temperature. The cells were then incubated with primary antibodies (rabbit polyclonal anti-CDNF 1:500, Sigma-Aldrich; and mouse monoclonal anti-CHOP 1:100, Santa Cruz Biotechnology) overnight at 4 °C. The cells were then washed with PBS and incubated with secondary antibodies (Alexa Fluor 488-conjugated IgG 1:1000; and Alexa Fluor 594-conjugated IgG 1:1000; Invitrogen) in PBS for 40 min at 37 °C. Images were captured using a Nikon 80i light microscope equipped with a CCD camera.

### 4.11. Western Blot

Neurons receiving different treatments or tissues from the cerebral cortex were harvested with TNE buffer (10 mM Tris, pH 8.0, 150 mM NaCl, 1 mM EDTA, 1% NP-40, 10% glycerol with protease inhibitors) or SDS lysis buffer (50 mM Tris, pH 8.0, 1% SDS). Lysates were centrifuged and processed for SDS-PAGE and transferred onto nitrocellulose memebranes. Western blot analysis was performed. Primary antibodies (anti-CDNF antibody 1:500, Sigma-Aldrich; anti-CHOP 1:100, Santa Cruz Biotechnology; anti-MANF antibody 1:500, Abcam, Cambridge, MA, USA; anti-eIF2α antibody 1:1000, Cell Signaling Technology; anti-p-eIF2α antibody 1:500, Abcam; anti-Tubulin 1:1000 and anti-actin 1:1000, Sigma-Aldrich) and horseradish peroxidase (HRP)-conjugated secondary antibodies (1:10,000 dilution; Millipore) were utilized.

### 4.12. Statistical Analysis

A comparison between the Bederson score in the control group and CDNF group was performed by applying the non-parametric Mann–Whitney U test. The statistical significance between cell viability and the expression level of CDNF and ER makers in primary neurons were analyzed by applying one-way ANOVA, which was followed by LSD post hoc analysis to compare means from several groups simultaneously. Other group differences were analyzed by Student’s *t*-test. Data are presented as the mean ± SD, and Two-tailed *p* values < 0.05 were considered statistically significant. Statistical analyses were performed using SPSS statistical program, version 19.0.

## 5. Conclusions

CDNF has neuroprotective effects on cerebral ischemia and on the OGD cell model, and the protective mechanism of CDNF may occur through the ER stress pathway. These findings suggest that CDNF and MANF may have similar protective mechanisms during strokes.

## Supplementary Materials

Supplementary materials can be found at http://www.mdpi.com/1422-0067/19/7/1905/s1.
