# Supplementary material for: Cerebral Dopamine Neurotrophic Factor (CDNF) Has Neuroprotective Effects against Cerebral Ischemia That May Occur through the Endoplasmic Reticulum Stress Pathway
Source: Int J Mol Sci. 2018 Jun 29;19(7):1905. doi: 10.3390/ijms19071905 (PMC6073452; doi:10.3390/ijms19071905)
Supplement: Supplementary file 1 [file ijms-19-01905-s001.pdf]

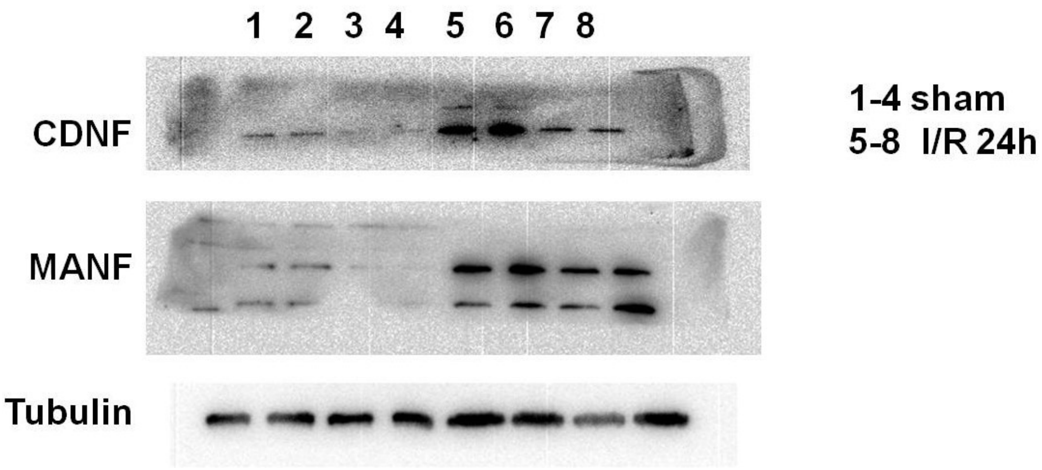

Figure S1. The representative full uncut Western blotting to show the expression of CDNF in the ischemic cerebral cortex, related to Figure 4C. The samples of the ischemic penumbra were collected at 2h ischemia/reperfusion 24 h. Tubulin was used as a loading control. 1-4 sham; 5-8 ischemia/perfusion. MANF as the positive control.

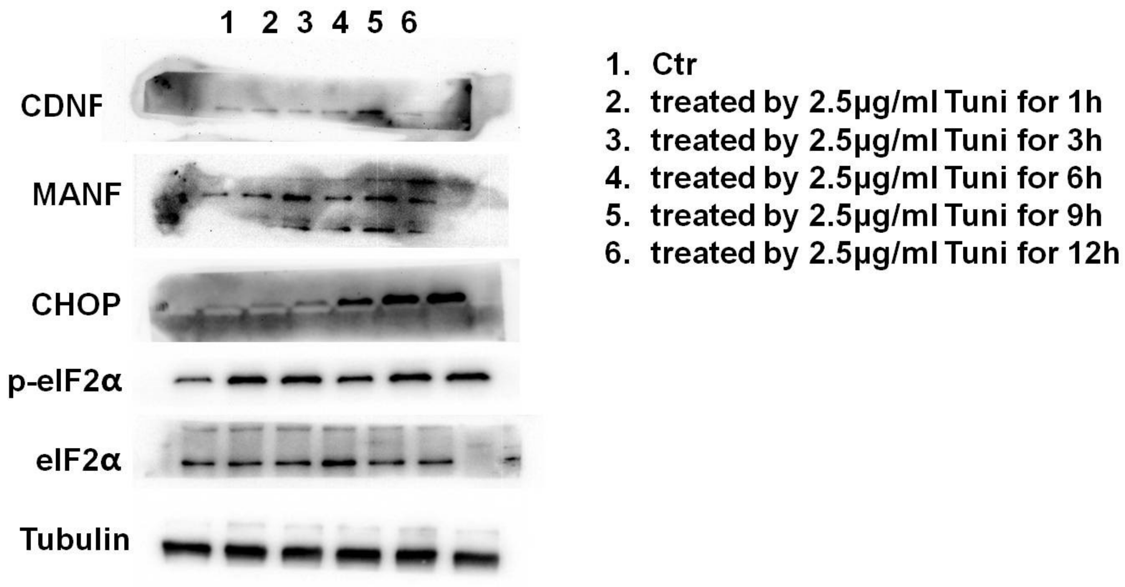

Figure S2. The representative full uncut Western blotting to show the expression of CDNF and ER markers induced by ER stress inducer tumicaycin, related to Figure 5B. The primary neurons were treated with 2.5 μg/mL of tunicamycin for 1, 3, 6, 9 and 12 h before being collected. Tubulin was used as a loading control; MANF as the positive control. 1, ctr; 2, 1h; 3, 3h; 4, 6h; 5, 9h; 6, 12h.
